# Is This Chatbot Safe and Evidence-Based? A Call for the Critical Evaluation of Generative AI Mental Health Chatbots

**DOI:** 10.2196/69534

**Published:** 2025-05-29

**Authors:** Acacia Parks, Eoin Travers, Ramesh Perera-Delcourt, Max Major, Marcos Economides, Phil Mullan

**Affiliations:** 1 Unmind Ltd, 140 Borough High St, London, SE1 1LB, United Kingdom, 1 2678798387

**Keywords:** GenAI, mental health, chatbot, ethics, evals

## Abstract

The proliferation of artificial intelligence (AI)–based mental health chatbots, such as those on platforms like OpenAI’s GPT Store and Character. AI, raises issues of safety, effectiveness, and ethical use; they also raise an opportunity for patients and consumers to ensure AI tools clearly communicate how they meet their needs. While many of these tools claim to offer therapeutic advice, their unregulated status and lack of systematic evaluation create risks for users, particularly vulnerable individuals. This viewpoint article highlights the urgent need for a standardized framework to assess and demonstrate the safety, ethics, and evidence basis of AI chatbots used in mental health contexts. Drawing on clinical expertise, research, co-design experience, and the World Health Organization’s guidance, the authors propose key evaluation criteria: adherence to ethical principles, evidence-based responses, conversational skills, safety protocols, and accessibility. Implementation challenges, including setting output criteria without one “right answer,” evaluating multiturn conversations, and involving experts for oversight at scale, are explored. The authors advocate for greater consumer engagement in chatbot evaluation to ensure that these tools address users’ needs effectively and responsibly, emphasizing the ethical obligation of developers to prioritize safety and a strong base in empirical evidence.

## A Call for the Critical Evaluation of Mental Health Chatbots

The internet is flooded with mental health resources, and one of the most common emerging formats is the artificial intelligence (AI) chatbot. A recent Forbes article examines the launch of OpenAI’s GPT store, which allows users to post chatbots for ready use by others, and found that many were intended for mental health advisory purposes; another 3 million or so general-purpose chatbots are not intended specifically for mental health purposes but would take on that role if prompted [1]. For example, a quick Google search for “Character.AI” and “therapist” yields a link to a Character.AI bot that says they have “been working in therapy since 1999… [are] a Licensed Clinical Professional Counselor (LCPC)... [and are] trained to provide EMDR treatment in addition to Cognitive Behavioral (CBT) therapies.” A small disclaimer at the bottom states, “This is A.I. and not a real person. Treat everything it says as fiction.” However, the boundary between reality and fiction can become quite blurry for consumers interacting with AI chatbots, as is illustrated by instances where deaths by suicide have been linked to chatbot usage [2].

This is particularly pertinent for chatbots which use Generative AI (GenAI). Although mental health chatbots have existed for some time, their increasing popularity is in part due to the rise of GenAI. In traditional chatbots, the user’s interaction with the bot is typically governed by an explicitly programmed set of rules for choosing between prewritten responses. GenAI chatbots, in contrast, are driven by powerful large language models (LLMs) that produce customized responses to each user message, guided by the instructions written in the “system prompt” provided to the LLM. Generative chatbots provide much greater flexibility at the cost of less predictable behavior.

The legality of such apps, when used for mental health, is questionable, as digital products that make medical claims, such as the ability to treat depression or anxiety, are considered medical devices in many countries. Medical devices are subject to requirements to show evidence of safety and effectiveness, as well as regulatory scrutiny. But the large majority of digital products that make these types of claims are not evaluated by regulatory bodies [3]. Somewhere in between “free for all” and “medical device” is a category of digital products that may provide advice responsibly without claiming they provide treatment. These chatbots can be considered “general mental health support” bots, as opposed to conversational AI chatbots, which have a specific purpose such as triage [4]. Examples include Ada [5], Chai [6], Elomia [7], Mindspa [8], Nuna [9], Serenity [10], Stresscoach [11], Woebot [12], Wysa [13], and Youper [14,15], as well as newer entrants Ebb (Headspace [16]) and Nova (Unmind [17]). Because these and other similar chatbots do not rise to the level of a medical device, regulatory bodies (eg the US Food and Drug Administration) do not govern the claims made about what the chatbots do. Consumers are therefore left to navigate this landscape without guidance on what makes a chatbot safe and effective. However, there is currently no legal, academic, or industry-agreed standard or method for doing this in a way that enables consumers to be meaningful, active collaborators in their own care.

We argue that companies producing AI mental health products intended for general use should demonstrate, in some systematic and objective way, that the products they provide to consumers are safe and deliver advice that is evidence-based. We argue that doing so is an ethical obligation to consumers, as well as something (quite rightly) expected of digital mental health interventions by both users and providers who recommend digital products. To empower consumers and the public to accurately assess the risks and benefits of using AI for self-care, there needs to be a clear, accessible framework for evidencing how the chatbot addresses the needs and concerns of the individual user. Such a framework will also need to be meaningful and acceptable to potential gatekeepers of access to AI, such as therapists referring patients to AI-based products or employer health benefits providers.

## What Criteria Should Generative, General Mental Health Chatbots Be Evaluated On?

Evaluating mental health–related chatbots is a particular challenge due to the sensitive nature of mental health, and the consequences of providing poor-quality responses to potentially vulnerable users discussing sensitive topics. Based on our shared experience in clinical practice, mental health research co-design and/or participatory involvement in research and building AI-powered products, and on the World Health Organization’s guidance on Ethics & Governance of Artificial Intelligence for Health (2024) [18], we propose that mental health AI chatbots should adhere to a version of the criteria outlined in Table 1.

Whatever criteria we use and whatever thresholds we set for expected performance of a chatbot, they should have real-world impact and reflect what matters most to users, including perceived relevance and usefulness, privacy and confidentiality [19], and human therapist personal attributes valued by consumers that may be replicable by AI chatbots, such as being respectful, confident, warm, and interested [20,21].

**Table 1.** Criteria for evaluating performance of an artificial intelligence–based mental health chatbot.

Criteria	Definition
Be ethical	Responses should benefit users while avoiding harm, be just and fair, promote user autonomy, and allow for transparent, informed understanding of their basis.
Be safe	Clear rules governing a chatbot’s behavior when there is a risk of physical or psychological harm to the user or to others must be set and adhered to. These should establish the chatbot’s remit, including signposting to external resources and not providing medical diagnosis or treatment or producing any outputs that would constitute use as a regulated medical device.
Be accessible	The chatbot should be accessible to the user, including support for the user’s native language where possible and appropriate accommodation for the user’s verbal comprehension skills.
Follow the evidence base	Responses should be grounded in the established scientific literature.
Apply core coaching skills	The chatbot should display strong conversational skills and apply conversational techniques including goal identification, alliance building, and empathetic inquiry.

## How Could Evaluation Be Implemented?

With the explosion in applications of GenAI, there is greater emphasis placed on “evals,” which are systematic approaches to evaluating whether the outputs of the AI system are appropriate for the task at hand before they are rolled out to users [22,23]. Evals will typically consist of a collection of test inputs to the AI system and criteria or scoring rules by which to evaluate the outputs. There are some scenarios where the accuracy of outputs may be evaluated directly, for instance, by comparing against a predefined target or using pattern matching. In other cases, for instance, in applications involving classification, data retrieval, or summarization, outputs can be compared against targets using statistical metrics such as precision and recall.

However, in many applications of GenAI, particularly those involving chatbots, there is no meaningful “right answer” for the chatbot to give. In these cases, we must instead evaluate outputs against a rubric or set of qualitative criteria. Criteria might include formatting features (eg, uses markdown), linguistic style (eg, level of formality), tone of voice (eg, level of warmth), or more abstract features (eg, shows empathy). This approach is used in the reinforcement learning phase of training modern AI LLMs, where models will generate multiple candidate responses to a given question, the preferred response is identified using predefined criteria, and this feedback is used to adjust the model to make such a response more likely [24,25], but is equally useful in evaluating models after training.

Evaluations against criteria can be performed either by human annotators or by additional AI systems. Expert human annotators can bring deep clinical expertise and nuanced understanding to their evaluations [25,26]. However, this approach is extremely resource-intensive and may suffer from unreliability or inconsistency, particularly when annotating large datasets [27]. An emerging alternative is the “LLM-as-a-judge” approach [28,29], where these evaluations are performed by an LLM. To work reliably, this approach requires an additional process of comparing LLM-generated evaluations against high-quality human evaluations, and modifying the instruction prompt used by the LLM to align and calibrate the human and AI judgements.

Writing criteria against which to evaluate AI-generated responses is a deceptively difficult task, requiring a deep understanding of the domain and the likely behaviours of both the users and the chatbot. It is increasingly recognized that the implicit criteria used by human annotators evolve as they are exposed to a greater variety of data [29]. It is considered best practice [29] to write these criteria iteratively, with expert judges continuously reviewing real user data alongside the previous generation of LLM-judged evals in order to produce criteria that better define how a chatbot should behave.

For chatbots, evals based on single interactions (a message and a response) may fail to capture important dynamics that emerge over multiple turns in a conversation. A promising approach is to use an additional AI system to play the role of the user interacting with the target chatbot in order to simulate multiturn “bot-to-bot” conversations. This approach has its challenges. If we intend to generalize from the chatbot’s responses in these simulated conversations to how the chatbot would respond in real interactions with humans, we must ensure that the messages from the simulated user are representative of the range of messages that would be sent by real users. Multiturn conversations can also go down many more diverging paths than single interactions; hence, a large number of simulated conversations under the same conditions may be needed to allow for the variance in outcomes.

## The Role of the Consumer

Much research to date has focused on using professional experts, not health care users, to evaluate chatbots. Although inconsistent, research has shown that coproduction of digital mental health interventions can improve their utility [30]. Similar to how there is a need for guidelines around user involvement in intervention development [31,32], we believe that the implementation of a critical evaluation framework for mental health AI chatbots would benefit from health care consumers not only contributing to the evaluation criteria but also being involved in rating chatbot conversations to calibrate the automated testing systems. Our viewpoint builds on previous work that has discussed issues around ensuring AI for consumers is safe, effective, and trustworthy [33,34]. This would ensure that health chatbots are evaluated in line with not only what previous research has demonstrated is important to consumers but also what is currently most relevant, given this technology is emergent. Furthermore, patients have a very different level of fluency with mental health concepts than the average researcher or practitioner, making their input particularly important in the development of mental health AI chatbots. A quote from an anonymous patient (interviewed March 13, 2025) highlights this:

*I use chatbots that are experts in all kinds of different therapeutic approaches. I get a lot out of them, but I’m also very aware that because I am well-versed in the therapeutic approaches they use, I’m able to ask them for the right things, in the right language. I recognize the concepts they are leveraging and find myself unconsciously staying within the bounds of what therapy is intended to do. I would never trust these chatbots in the hands of the average consumer. There are so many ways to misunderstand meaning or offer the wrong thing if the language of the input is ‘wrong’.*

In other words, practitioners and software developers emulating patients are not enough to capture the many ways that a therapeutic chatbot could err—naturalistic patient use will unearth new use cases and reveal new pitfalls. A number of recent papers provide models for taking a participatory approach to designing and testing GenAI tools.

## Conclusions

Digital mental health is rife with products that are unhelpful at best and compromise consumer safety at worst. In order to realize the potential of GenAI for mental health, it is recognized that all stakeholders need to be involved in its development and regulation [34]. We have argued for the importance of evaluating GenAI mental health chatbots, even in a nonregulated context, objectively, with a common set of criteria that can provide guidance for consumers and practitioners on which products are safe and evidence-based. We provide some suggestions to start and highlight some of the key challenges to implementing those suggestions. By involving consumers in the evaluation process, and addressing their needs during development, the true promise of GenAI can be realized for all health care users. At the same time that we push for more rigorous evaluation and regulation of GenAI-based digital mental health products, we must also keep in mind the urgent need for such products, and the potential cost of hindering progress. A patient cited in the Medicines & Healthcare products Regulatory Agency (MHRA) research report on digital mental health technology says, “I think apps are likely to be safer than the range of side effects present in many meds” [35]. For some patients, digital mental health products may be appealing in a way that other forms of treatment are not, such that they will not seek in-person care if digital options are not available. Another patient in the MHRA report notes, “People may find it easier to write how they are feeling rather than struggling to find the words or sentences” [35]. Further, as the earlier anonymous patient highlighted to us, “The alternative [to using GenAI therapy] for me is to receive nothing, and that’s the norm. The majority of patients receive no care at all.” So, even as we work to keep digital products safe and ensure their effectiveness, we must also be mindful that the need for these solutions is high, and the risk of not making digital solutions available may be higher than the risks of offering them.

## Abbreviations

AI: artificial intelligence
GenAI: generative artificial intelligence
LLM: large language model
MHRA: Medicines & Healthcare products Regulatory Agency
